# Improving the Efficiency of CRISPR Ribonucleoprotein-Mediated Precise Gene Editing by Small Molecules in Porcine Fibroblasts

**DOI:** 10.3390/ani14050719

**Published:** 2024-02-25

**Authors:** Yunjing Zhao, Xinyu Li, Chang Liu, Chaoqian Jiang, Xiaochen Guo, Qianqian Xu, Zhi Yin, Zhonghua Liu, Yanshuang Mu

**Affiliations:** 1 Key Laboratory of Animal Cellular and Genetic Engineering of Heilongjiang Province, Northeast Agricultural University, Harbin 150030, China; j18845832622@163.com (Y.Z.); lxy17372787325@163.com (X.L.); liuchang8799@outlook.com (C.L.); jiangchaoqianneau@163.com (C.J.); s190901057@neau.edu.cn (X.G.); xuqianqian@neau.edu.cn (Q.X.); zyin@neau.edu.cn (Z.Y.); 2 College of Life Science, Northeast Agricultural University, Harbin 150030, China

**Keywords:** pig, CRISPR/Cas9, small molecule, precise gene editing, HDR

## Abstract

**Simple Summary:**

Clustered regularly interspaced short palindromic repeats (CRISPR) ribonucleoprotein (RNP)-mediated precise genome editing can modify a specific targeted gene to establish cell models and improve animal traits, thus playing a significant role in animal breeding. However, the efficiency of precise gene editing is low, which limits its widespread use. To overcome this problem in porcine cells, we used small molecules to improve the efficiency of CRISPR RNP-mediated precise genome editing in porcine fetal fibroblasts (PFFs). We demonstrated that a single small molecule including L-189, NU7441, SCR7, L755507, RS-1, and Brefeldin A increased the efficiency of homology-directed repair (HDR) during precise gene editing in PFFs, respectively. These results provided methodological support for the establishment of an efficient and precise gene editing system in pigs.

**Abstract:**

The aim of this study was to verify whether small molecules can improve the efficiency of precision gene editing using clustered regularly interspaced short palindromic repeats (CRISPR) ribonucleoprotein (RNP) in porcine cells. CRISPR associated 9 (Cas9) protein, small guide RNA (sgRNA), phosphorothioate-modified single-stranded oligonucleotides (ssODN), and different small molecules were used to generate precise nucleotide substitutions at the insulin (INS) gene by homology-directed repair (HDR) in porcine fetal fibroblasts (PFFs). These components were introduced into PFFs via electroporation, followed by polymerase chain reaction (PCR) for the target site. All samples were sequenced and analyzed, and the efficiencies of different small molecules at the target site were compared. The results showed that the optimal concentrations of the small molecules, including L-189, NU7441, SCR7, L755507, RS-1, and Brefeldin A, for in vitro-cultured PFFs’ viability were determined. Compared with the control group, the single small molecules including L-189, NU7441, SCR7, L755507, RS-1, and Brefeldin A increased the efficiency of HDR-mediated precise gene editing from 1.71-fold to 2.28-fold, respectively. There are no benefits in using the combination of two small molecules, since none of the combinations improved the precise gene editing efficiency compared to single small molecules. In conclusion, these results suggested that a single small molecule can increase the efficiency of CRISPR RNP-mediated precise gene editing in porcine cells.

## 1. Introduction

As an important livestock in agriculture, pigs are high-quality meat sources for people in many countries and have been genetically modified to present desirable traits of economic importance [1]. Precise gene editing can obtain gene modifications at specific genome loci to improve the traits of pigs, such as the myostatin (MSTN) gene for meat production and CD163 gene for porcine reproductive and respiratory syndrome virus (PRRSV) resistance [2,3]. Precise gene editing can also establish a cellular model for studying the mechanism of single nucleotide polymorphism (SNP)-related loci, which are significantly associated with production traits in genetic breeding [4].

CRISPR/Cas9-mediated gene editing intentionally produces site-specific DNA double strand breaks (DSBs) to modify the genomic sequence [5,6]. The DSB is repaired by endogenous DNA repair pathways, either nonhomologous end joining (NHEJ) or homology-directed repair (HDR). The predominant and error-prone NHEJ pathway often results in small nucleotide insertions or deletions that can be used to construct knockout alleles [5]. Alternatively, HDR activity can result in precise modification, incorporating exogenous DNA fragments into the cut site [6].

The DNA template used in HDR can be double-stranded DNA or single-stranded DNA. SsODNs represent an attractive option as an HDR donor template for small, controlled mutations or insertions. Today, ssODNs are widely used in biotechnology [7], cellular and animal model generation [8], and therapeutic applications using Fok1-based nucleases and CRISPR/Cas9 [9,10]. The main advantages of ssODN templates include affordable and scalable production, relatively low toxicity, and a reduced risk of integration by ligation mechanisms. Repair from dsDNA donors is relatively inefficient in most cell types and is assumed to utilize a repair mechanism that parallels meiotic homologous recombination [11]. In contrast, SSTR is highly effective in human cells (>20% of alleles) and broadly conserved among metazoans [12]. While ssODN can be cotransfected with nuclease in the form of plasmid DNA [13] and mRNA [14], the most promising results have been achieved with ssODN paired with Cas9 RNP [15,16]. In a comprehensive study by Schubert et al., knock-in activity mediated by Cas9 RNP with HDR efficiency was tested at 254 genomic loci in Jurkat cells and 239 genomic loci in HAP1 cells, achieving up to a 50% HDR rate for selected experimental conditions and genomic loci [16].

Multiple strategies have been developed to increase the efficiency of HDR in mammalian systems [17]. Cell-permeable small molecules are a promising approach to enhance CRISPR/Cas9 genome-editing efficiency owing to their intrinsic properties, such as reversibility and low immunogenicity. Furthermore, small molecules are easier to store and produce on a commercial scale compared with the production and purification of proteins. Several small molecules have been identified to modulate CRISPR–Cas9-induced genome editing [18]. The mechanisms of action of these small molecule compounds include improving the efficiency of precise gene editing by inhibiting nonhomologous end joining; enhancing precise gene editing by using a DNA ligase IV inhibitor; increasing the efficiency of precise gene editing by promoting homologous recombination; increasing homologous directed repair by synchronizing the cell cycle; and improving the efficiency of homologous directed repair by an unknown mechanism [19]. Although chemical strategies have great applications in stem cell biology and regenerative medicine [20], it remains to be confirmed which small molecule compounds can improve the efficiency of ssDNA template-mediated precise gene editing methods and how much the recombination efficiency can be improved [17].

Porcine insulin gene (INS) is mainly expressed in the pancreas and plays an important role in carbohydrate and lipid metabolism [21]. Pig insulin differs from human insulin by one amino acid (alanine in pigs and threonine in humans) at the carboxy terminus of the B chain (i.e., position B30) at the protein level [22]. The main objective of this study is to perform precise gene editing on this base site in the coding region of INS gene, and generate base substitution in pig cells.

To more accurately determine which small molecules can improve the efficiency of ssDNA donors in mediating CRISPR RNP-induced homologous recombination in porcine fetal fibroblasts, we investigated the effects of six small molecules, namely L-189, NU7441, SCR7, L755507, RS-1, and Brefeldin A, on CRISPR/Cas9-mediated HDR with ssDNA donors.

## 2. Materials and Methods

### 2.1. Cell Culture

The porcine fetal fibroblasts (PFFs) used in this study were obtained from Large White pigs at the Northeast Agricultural University Embryo Engineering Laboratory Experimental Pig Base. All animal-related procedures followed the guidelines set forth by the Institutional Animal Care and Use Committees (IACUCs) of Northeast Agriculture University. The PFF culture method has been previously published [23]. Briefly, PFFs were isolated from a 33-day-old foetus and cultured in Dulbecco’s modified Eagle medium (DMEM, Gibco, New York, NY, USA) supplemented with 15% foetal bovine serum (FBS, HyClone, New York, NY, USA), 1% penicillin–streptomycin (Gibco, New York, NY, USA), 1% nonessential amino acids (Gibco, New York, NY, USA), and 2 mmol/L L-glutamine (Sigma, New York, NY, USA). The cells were passaged every two days using 0.25% trypsin-EDTA (Gibco, New York, NY, USA). The small molecules L-189 (CAS No. HY-15588), NU7441 (CAS No. HY-11006), SCR7 (CAS No. HY-12742), L755507 (CAS No. HY-19334), RS-1 (CAS No. HY-19793), and Brefeldin A (CAS No. HY-16592) were sourced from Med Chem Express Company (Monmouth Junction, NJ, USA). The small molecules were added in cell culture medium after CRISPR RNP electroporation at different concentrations.

### 2.2. Design of gRNAs and ssODNs

Pig gene INS was chosen to design CRISPR targets against, with one sgRNA per INS gene. The sgRNA sequence was 5-CAC GCC CAA GGC CCG TCG GG-3. The PAM site was AGG. To introduce the base modification, 116 bp ssODNs as donor DNA were introduced into the target using HDR-mediated precise gene editing. The ssODN sequences are shown in Supplementary Table S1. The sgRNA and ssODN templates with thiomodification were synthesized by GenScript (Nanjing, China).

### 2.3. Oligonucleotide and Ribonucleoprotein Electroporation

After trypsin digestion of the PFFs, the digestion was terminated by adding twice the volume of pancreatic enzyme culture medium. The cells were counted by the trypan blue dye exclusion assay. Briefly, the cells were diluted in suspension with 0.4% trypan blue solution in 1:1 ratio, the cells were counted in four 1 × 1 mm squares of one chamber of hemocytometer under a microscope, and 1 × 10^6^ cells were used. To prepare the ribonucleoprotein (RNP) mixture, for 1 × 10^6^ cells, we premixed 10 μg of Cas9 protein (GenScript, Nanjing, China) with 100 pmol of sgRNA (GenScript, Nanjing, China) being added for 10 min at room temperature. Then, we added 200 pmol of ssODN (GenScript, Nanjing, China) and supplemented it to a final volume of 20 μL with OptiMEM (Thermo, Shanghai, China). We then resuspended the cell pellet containing 1 × 10^6^ cells with the RNP mixture, gently pipetted the mixture to obtain a uniform cell suspension, and transfered it to the electroporation cuvette. The cells were electroporated and transfected according to the established procedure using an electroporation instrument. Following electroporation, the transfected cells were seeded into appropriate petri dishes and cultured as needed.

### 2.4. Cell Viability Assay

To evaluate cell viability, the Enhanced Cell Counting Kit-8 (Beyotime, Shanghai, China) was used with the following steps: Seed cells into a 96-well plate at a density of approximately 50%. After 24 h, replace the culture medium with small molecule-containing medium and incubate for an additional 24 h. Add 20 μL of the enhanced CCK-8 solution to each well and incubate in a cell culture incubator for 0.5–4 h. The absorbance was measured at 450 nm using a microplate reader. The cell viability was calculated using the A450 value obtained.

### 2.5. DNA Extraction and PCR

Genomic DNA was extracted from harvested cells with a Universal Genomic DNA Extraction Kit (Takara, Dalian, China) using standard procedures. Then, 200–300 bp DNA fragments containing the targeted region were amplified by high-fidelity DNA polymerase, and the DNA fragments were purified from gels by a gel extraction kit (Takara, Dalian, China). The primer sequences were 5′-CCG GCC CAG GCC TTC GTG AAC CA-3′ and 5′-GGC TTC TCG AGC GGG ACC GGG-3′.

### 2.6. Deep Sequencing (Deep-Seq) and Analysis

The PCR amplicons were deep sequenced commercially (Annoroad Gene Technology, Hangzhou, China). Illumina-compatible adapters with unique barcodes were ligated onto each sample during library construction. Libraries were pooled in equimolar concentrations for multiplexed sequencing on the Illumina MiSeq platform with 2 × 150 run parameters. Indel rates were determined by the CRISPResso2 tool [24].

### 2.7. Statistical Analysis

SPSS 16.0 Statistical Software was used to conduct statistical analysis (SPSS, Inc., Chicago, IL, USA). All results are represented as the mean ± SE of at least three distinct repeated experiments. The *t*-test was used to compare the differences. Statistical significance of the difference was defined as a value of *p* < 0.05.

## 3. Results

### 3.1. The Effect of Different Concentrations of Small Molecules on Cell Viability

**Figure 1:**
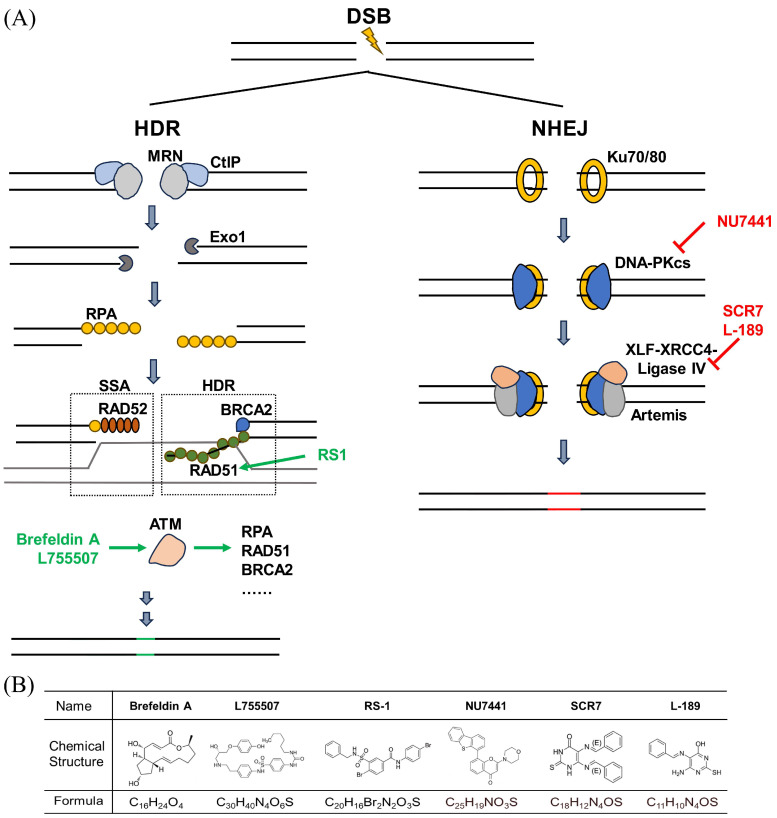
Small molecules described to target key proteins of NHEJ and HDR. (**A**) Proteins are labelled with black text, and inhibitors and enhancing small molecules are marked in red and green, respectively. NU7441, L-189, and SCR7 have been described to inhibit DNA-PK and DNA ligase IV, respectively. RS-1 has been described to enhance RAD51. L755507 and Brefeldin A have been described to enhance ATM. For simplicity, some proteins and protein interactions are not depicted. (**B**) Molecular formula and chemical structure of six small molecules.

To verify small molecules that can improve the efficiency of ssODN-mediated CRISPR/Cas9 precision editing in PFFs, we searched the literature and relevant databases (ChEMBL) [25] and identified the molecules that interact with repair proteins listed in the REPAIRtoire database [26]. Six small molecules were tested. Three small molecules, including L-189, NU7441, and SCR7, can block NHEJ/alternative NHEJ. Three other small molecules, L755507, RS-1, and Brefeldin A, can enhance the efficiency of CRISPR-mediated homologous recombination repair (Figure 1A,B).

**Figure 2:**
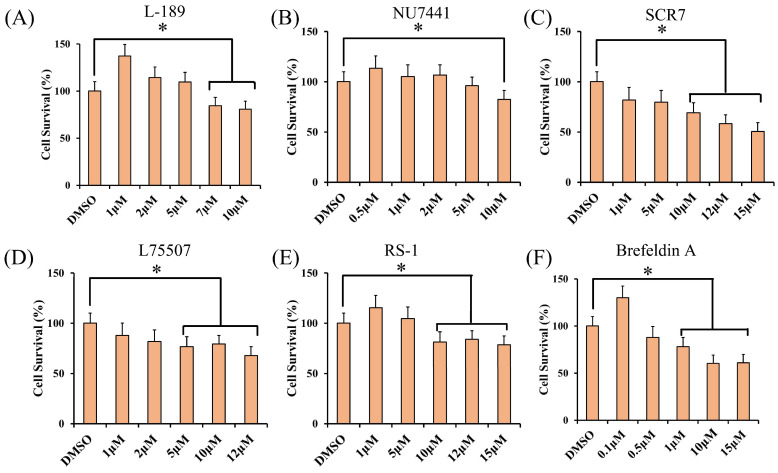
Effects of the different small molecules on the cell survival in PFFs. Resazurin assay for cell survival in PFF cells 3 days after treatment with different concentrations of (**A**) L-189, (**B**) NU7441, (**C**) SCR7, (**D**) L755507, (**E**) RS-1, and (**F**) Brefeldin A. Error bars show the SEM of three replicates for PFF cells. * *p* < 0.05.

To examine the toxic effect of small molecules on PFFs, different concentrations of six small molecules were used to conduct cytotoxicity experiments, and the needed concentrations of small molecules for precise gene editing were determined. L-189 had no effect on cell viability when the concentration was between 1 and 5 μM, but the cell viability rapidly decreased after 5 μM (Figure 2A). There was no change in cell viability for NU7441 when the concentration was between 0.5 and 2 μM, and it began to decrease after 2 μM (Figure 2B). The addition of SCR7 caused a decrease in cell viability, and when the concentration was 5 μM, the cell viability decreased to approximately 85% (Figure 2C). The cell viability began to decrease with the addition of L755507, and when the concentration was 5 μM, the cell viability decreased to approximately 85% (Figure 2D). When the RS-1 concentration was between 1 and 5 μM, there was no change in cell viability, but it decreased to approximately 90% when the concentration was 10 μM (Figure 2E). Brefeldin A had no effect on cell viability when the concentration was 0.1 μM, but the cell viability began to decrease to approximately 85% when the concentration was 0.5 μM (Figure 2F).

The required concentrations of small molecules for precise gene editing were determined through cytotoxicity experiments and a literature review, which were based on the effect of small molecule concentrations on cell viability and CRISPR-mediated gene editing efficiency [19,27]: L-189 at 5 μM; NU7441 at 1 μM; SCR7 at 10 μM; L755507 at 5 μM; RS-1 at 10 μM; and Brefeldin A at 0.5 μM (Figure 2).

### 3.2. The Effect of Individual Small Molecules on the Efficiency of CRISPR RNP Mediated Precise Genome Editing

**Figure 3:**
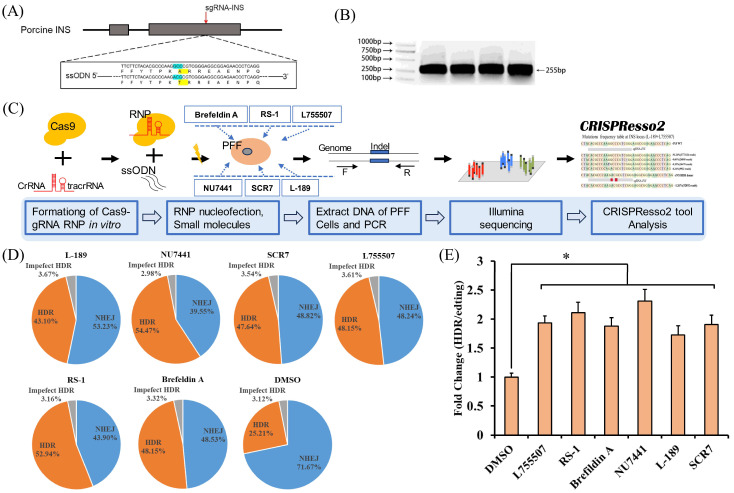
Effects of different small molecules on the efficiency of HDR in PFF. (**A**) Schematic of INS gene structure and sgRNA-INS target site. Replacement base and amino acid sites are indicated in green and yellow, respectively. (**B**) PCR amplification of the INS gene target sequence. (**C**) Schematic of the evaluation of the genome editing efficiencies of INS with Cas9 protein in PFF cells by small molecules. (**D**) Genome editing efficiencies of INS with Cas9 protein in PFF cells with different small molecules. (**E**) Histogram showing genome editing efficiencies of INS with Cas9 protein in PFF cells treated with the different small molecules. Small molecules were added for 3 days after editing. HDR, homology-directed repair; Imperfect HDR, homology-directed repair with indels; NHEJ, nonhomologous end joining. Error bars show the SEM of three replicates for PFFs. * *p* < 0.05.

The target gene for this experiment was INS, and the sgRNA-INS was designed and synthesized in vitro (Figure 3A). The INS gene target sequence can be obtained through PCR amplification (Figure 3B). The premixed RNP and single-stranded oligodeoxynucleotide template ssODN-INSA54T-M were cotransfected into porcine fibroblasts via electroporation, with different small molecules being added during the culturing. After 2 days of culturing, the cells were collected to detect the gene editing efficiency (Figure 3C and Figure S1).

The sequencing analysis results showed that the percentage of correctly edited cells via HDR were 25.21% in the DMSO group, 43.10% in the L-189 group, 57.47% in the NU7441 group, 47.64% in the SCR7 group, 48.15% in the L755507 group, 52.94% in the RS-1 group, and 48.15% in the Brefeldin A group (Figure 3D). Compared to the DMSO group, the small molecules L-189, NU7441, SCR7, L755507, RS-1, and Brefeldin A increased the precise gene editing efficiency by 1.71-fold, 2.28-fold, 1.89-fold, 1.91-fold, 2.10-fold, and 1.87-fold, respectively (Figure 3E).

### 3.3. The Effect of Combining of Two Small Molecules on the Efficiency of CRISPR RNP Mediated Precise Genome Editing

**Figure 4:**
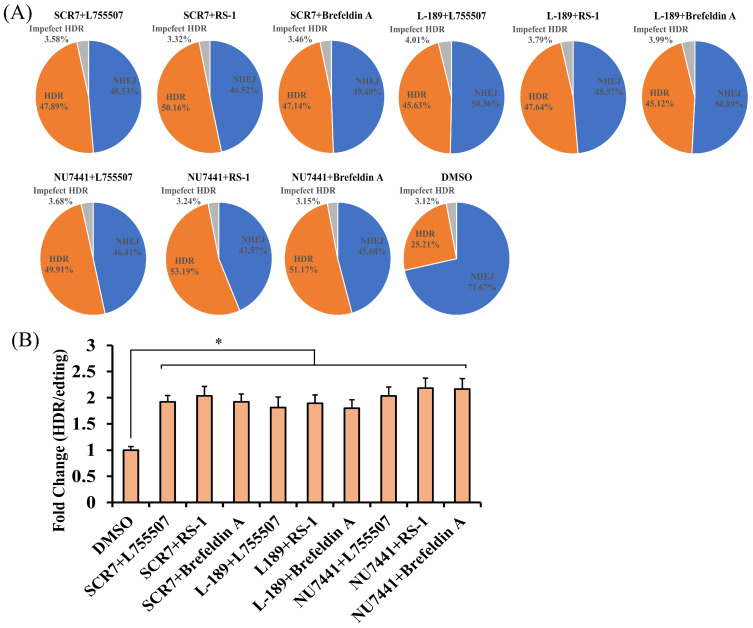
Effects of HDR on the efficiency of HDR in PFF by combining SCR7 + L755507, SCR7 + RS-1, and SCR7 + Brefeldin A. (**A**) Genome editing efficiencies of INS with Cas9 protein in PFF cells with the combination of two small molecules, SCR7 + L755507, SCR7 + RS-1, SCR7 + Brefeldin A, L-189 + L755507, L-189 + RS-1, L-189 + Brefeldin A, NU7441 + L755507, NU7441 + RS-1, and NU7441 + Brefeldin A. (**B**) Histogram showing genome editing efficiencies of INS with Cas9 protein in PFF cells treated with the combination of two small molecules, SCR7 + L755507, SCR7 + RS-1, SCR7 + Brefeldin A, L-189 + L755507, L-189 + RS-1, L-189 + Brefeldin A, NU7441 + L755507, NU7441 + RS-1, and NU7441 + Brefeldin A. Small molecules were added for 3 days after editing. HDR, homology-directed repair; Imperfect HDR, homology-directed repair with indels; NHEJ, nonhomologous end joining. Error bars show the SEM of three replicates for PFFs. * *p* < 0.05.

To investigate the effect of the combined use of small molecules on CRISPR-mediated precise gene editing, double small molecule combinations were designed. The combinations of SCR7 with different NHEJ inhibitors were as follows: SCR7 (10 μM) + L755507 (5 μM), SCR7 (10 μM) + RS-1 (10 μM), and SCR7 (10 μM) + Brefeldin A (0.5 μM). The sequencing analysis results showed that the percentages of correctly edited cells via HDR were 47.89% in the SCR7 + L755507 group, 50.16% in the SCR7 + RS-1 group, and 47.14% in the SCR7 + Brefeldin A group (Figure 4A and Figure S2). Compared to the DMSO group, the small molecule combinations SCR7 + L755507, SCR7 + RS-1, and SCR7 + Brefeldin A resulted in increased precise gene editing efficiencies by 1.90-fold, 1.99-fold, and 1.87-fold (Figure 4B).

The combinations of L-189 with different NHEJ inhibitors were as follows: L-189 (5 μM) + L755507(5 μM), L-189 (5 μM) + RS-1 (10 μM), and L-189 (5 μM) + Brefeldin A (0.5 μM). The sequencing analysis results showed that the percentage of correctly edited cells via HDR were 45.63% in the L-189 + L755507 group, 47.64% in the L-189 + RS-1 group, and 45.12% in the L-189 + Brefeldin A group (Figure 4A and Figure S2). Compared to the DMSO group, the small molecule combinations L-189 + L755507, L-189 + RS-1, and L-189 + Brefeldin A increased the precise gene editing efficiencies by 1.81-fold, 1.89-fold, and 1.79-fold (Figure 4B).

The combinations of NU7441 with different NHEJ inhibitors were as follows: NU7441 (1 μM) + L755507 (5 μM), NU7441 (1 μM) + RS-1 (10 μM), and NU7441 (1 μM) + Brefeldin A (0.5 μM). The sequencing analysis results showed that the percentages of correctly edited cells via HDR were 49.91% in the NU7441 + L755507 group, 53.19% in the NU7441 + RS-1 group, and 51.17% in the NU7441 + Brefeldin A group (Figure 4A and Figure S2). Compared with the DMSO group, the small molecule NU7441 + L755507, NU7441 + RS-1, and NU7441 + Brefeldin A combinations increased the precision gene editing efficiencies by 1.98-fold, 2.11-fold, and 2.03-fold (Figure 4B).

## 4. Discussion

Despite the versatility and stable use of CRISPR/Cas9, some issues limit its application. For example, the precise gene modification efficiency mediated by CRISPR/Cas9 is lower in several mammalian cells. The main reason for the low efficiency of precision gene editing is that HDR and NHEJ compete in the DSB repair process. One of the important requirements for precise gene editing using CRISPR is to improve the efficiency of HDR in a simple and repeatable way. Improving HDR’s efficiency not only facilitates the use of precise gene editing in therapeutic environments but also facilitates biomedical and drug discovery research from model generation to disease mutation [19]. With more understanding of how the HDR repair pathway is suppressed in the G1 phase of the cell cycle and stimulated in the S/G2 phases, methods can be developed to selectively disrupt error-prone repair pathways, particularly NHEJ, thereby promoting precise HDR; directly promoting HDR repair over NHEJ; or providing a favourable cellular environment for HDR (S/G2 phase, pairing Cas9-induced DSBs with DNA template) [28].

Compared to previous studies targeting factors in the repair pathway or building related Cas9 fusion proteins, the advantages of HDR methods that use small molecules to direct DSB repair pathways lie in their ease of application in cell lines, reversibility, and rapid mode of action [29]. Therefore, the use of small molecule compounds to enhance CRISPR/Cas9 activity seems to be a promising approach. In this study, NU7441 inhibited DNA-PKcs, a key factor involved in the DNA NHEJ repair pathway [30]. L-189 and SCR7 are small molecule drugs that inhibit Ligase IV, another key factor involved in the DNA NHEJ pathway [31]. RS-1 is a small molecule drug that enhances RAD51, a key factor involved in the DNA HDR repair pathway [32]. L-755507 can improve the efficiency of CRISPR-mediated homologous recombination repair [33]. Brefeldin A is a small molecule drug that inhibits cell transport from the endoplasmic reticulum to the Golgi apparatus, that improves the efficiency of homologous recombination repair, and that is an enhancer of CRISPR-mediated HDR [33,34]. Therefore, using small molecules to improve CRISPR-mediated precise gene editing remains the best choice currently.

Comparing networks of genes involved in HDR from different donor templates (ssDonor vs. plasmid dsDonor) revealed that many DNA repair factors are shared between SSTR and HR [6,11,12,26]. It was noted that the repair proteins needed for SSTR in screening human cell lines revealed a very different set of necessary repair proteins, specifically multiple components of the Fanconi anaemia (FA) pathway [35]. The FA pathway is primarily associated with repairing interstrand crosslinks in the genome but also with protecting stalled replication forks [36]. In this study, small molecules such as L-189, SCR7, and NU7441 inhibited nonhomologous end joining in DNA repair processes, while RS-1, Brefeldin A, and L755507 enhanced the homologous recombination repair process in DNA repair. These small molecules also improve the efficiency of CRISPR-mediated precise gene editing with ssDonor. This is because the early steps of HDR may be similar for the templated repair of a Cas9 break with ssDonor or dsDonor, but the downstream stability and incorporation of different donor templates requires different factors. Future work could expand this platform to genome-wide screens or focus more narrowly on the FA pathway to determine whether different FA subcomplexes act specifically during SSTR or HR [35].

The general link between HDR stimulation and the cell cycle illustrates that HDR-boosting treatments can be a balancing act between desired and undesired outcomes. It is tempting to wish for a treatment that increases HDR to 100% efficiency, no matter the mechanism. Nevertheless, the combination of HDR-promoting small molecules with different modes of action may be a way to overcome the cell-type and gene-specificity limitations of single inhibitors. Furthermore, small molecule combinations that act on both HDR and NHEJ pathways simultaneously are more likely to be a method for improving the efficiency of precise gene editing. However, it was found that the combined treatment of SCR7 and RS-1 in MCF7 cells resulted in a slight but not significant increase in the gene editing efficiency of each compound [37]. The combination therapy of SCR7 and RS-1 in zebrafish also failed to demonstrate a significant increase over single molecule use [38]. In this study, small molecule combinations did not show efficiencies that were higher than the highest efficiency obtained when two small molecules were used alone. This suggests that dual targeting (inhibiting NHEJ while increasing HDR) may have strong limitations. The microtubule poison nocodazole boosts HDR in some contexts via G2/M arrest but also increases aneuploidy and the frequency of failed mitoses. Similarly, inappropriately activating DNA repair during mitosis leads to chromosome fusions [39]. A recent study defined a combination of four drugs called “CRISPY”, two known single-drug enhancers (NU7026 and trichostatin A) and two drugs with limited and inconsistent effects on gene editing efficiency when used alone (MLN4924 and NSC15520), which increased the gene editing efficiency by 7.2-fold [40]. This is also an important direction for future research.

Although many small molecules have been validated in animal models, their use has not yet become a standard procedure for CRISPR genome editing; this may be related to adverse reactions that small molecules may cause, such as off-target mutations, immune reactions, chromosomal translocations, and genetic toxicity. For instance, inhibitors and enhancers of proteins that play a critical role in the DNA damage response can improve the genome editing efficiency of CRISPR/Cas9, but they may also affect off-target effects related to members of the protein family [10,41]. Small molecules targeting CRISPR/Cas9 exhibit cell type- and cell state-dependent effects, which may be due to the repair signal response of DNA damage [42]. In addition to classical NHEJ (cNHEJ) and HDR, other DNA repair pathways are also essential for the DNA damage response, especially in cNHEJ- and HDR-defective cells. For example, single-strand annealing (SSA), break-induced replication (BIR), and microhomology-mediated template switching have complementary roles in DSB-induced repair and are always considered error-prone pathways, which may lower the efficiency of HDR [43].

The advantages of using small molecules to pharmacologically guide DSB repair pathway selection towards HDR methods include their ease of application in cell lines, reversibility, and fast action mode. Therefore, small molecules are a promising method for precisely controlling and enhancing CRISPR/Cas9 genome editing, especially when combined with other methods. For example, CRISPR/Cas9-mediated genome editing treated with small molecules exhibits different enhancement or inhibition effects when delivered by the electroporation, lipid transfection, or nuclear transfection of Cas9-RNP complexes [15,41]. Meanwhile, small molecules can effectively regulate CRISPR/Cas9 activity in various cell types and other CRISPR/Cas-based systems (such as CRISPR/Cpf1, CBE) and may become a major editing tool in the future [44]. However, more work is needed to reveal the precise interaction between small molecules and Cas9 endonuclease and to expand the application of small molecules in CRISPR/Cas9 gene editing.

## 5. Conclusions

In conclusion, this study validated the small molecules L-189, NU7441, SCR7, L755507, RS-1, and Brefeldin A, which can improve the precision of CRISPR RNP-mediated precise gene editing in pig fibroblasts. The combination of two small molecules is not significantly different from the addition of a single small molecule. These results provide methodological support for the establishment of new strategies to increase the precision and efficacy of gene editing workflows in PFFs.

## Data Availability

The authors declare that all data supporting the findings of this study are available within the paper and its supplemental information files.

## Supplementary Materials

The following supporting information can be downloaded at: https://www.mdpi.com/article/10.3390/ani14050719/s1, Figure S1: The deep sequence results of INS gene precise editing in PFF cells treated with single small molecule L-189, NU7441, SCR7, L755507, RS-1 and Brefeldin A; Figure S2: The deep sequence results of INS gene precise editing in PFF cells treated with the combination of two small molecules, L-189 + L755507, L-189 + RS-1, L-189 + Brefeldin A, SCR7 + L755507, SCR7 + RS-1, SCR7 + Brefeldin A, NU7441 + L755507, NU7441 + RS-1, and NU7441 + Brefeldin A; Table S1: ssODN synthesized in this paper.

## References

[1] Yang H., Wu Z. (2018). Genome Editing of Pigs for Agriculture and Biomedicine. Front. Genet..

[2] Song R.G., Wang Y., Zheng Q.T., Yao J., Cao C.W., Wang Y.F., Zhao J.G. (2022). One-step base editing in multiple genes by direct embryo injection for pig trait improvement. Sci. China-Life Sci..

[3] Xu K., Zhou Y.R., Mu Y.L., Liu Z.G., Hou S.H., Xiong Y.J., Fang L.R., Ge C.L., Wei Y.H., Zhang X.L. (2020). CD163 and pAPN double-knockout pigs are resistant to PRRSV and TGEV and exhibit decreased susceptibility to PDCoV while maintaining normal production performance. eLife.

[4] Xiang G.H., Ren J.L., Hai T., Fu R., Yu D.W., Wang J., Li W., Wang H.Y., Zhou Q. (2018). Editing porcine regulatory element improved meat production in Chinese Bama pigs. Cell Mol. Life Sci..

[5] Chang H.H.Y., Pannunzio N.R., Adachi N., Lieber M.R. (2017). Non-homologous DNA end joining and alternative pathways to double-strand break repair. Nat. Rev. Mol. Cell Biol..

[6] Xue C.Y., Greene E.C. (2021). DNA Repair Pathway Choices in CRISPR-Cas9-Mediated Genome Editing. Trends Genet..

[7] Saha S.K., Saikot F.K., Rahman M.S., Jamal M.A.M., Rahman S.M.K., Islam S.M.R., Kim K.H. (2019). Programmable Molecular Scissors: Applications of a New Tool for Genome Editing in Biotech. Mol. Ther.-Nucleic Acids.

[8] Remy S., Chenouard V., Tesson L., Usal C., Menoret S., Brusselle L., Heslan J.M., Nguyen T.H., Bellien J., Merot J. (2017). Generation of gene-edited rats by delivery of CRISPR/Cas9 protein and donor DNA into intact zygotes using electroporation. Sci. Rep..

[9] Chen F.Q., Pruett-Miller S.M., Huang Y.P., Gjoka M., Duda K., Taunton J., Collingwood T.N., Frodin M., Davis G.D. (2011). High-frequency genome editing using ssDNA oligonucleotides with zinc-finger nucleases. Nat. Methods.

[10] Di Stazio M., Foschi N., Athanasakis E., Gasparini P., d’Adamo A.P. (2021). Systematic analysis of factors that improve homologous direct repair (HDR) efficiency in CRISPR/Cas9 technique. PLoS ONE.

[11] Fu Y.W., Dai X.Y., Wang W.T., Yang Z.X., Zhao J.J., Zhang J.P., Wen W., Zhang F., Oberg K.C., Zhang L. (2021). Dynamics and competition of CRISPR-Cas9 ribonucleoproteins and AAV donor-mediated NHEJ, MMEJ and HDR editing. Nucleic Acids Res..

[12] Gallagher D.N., Haber J.E. (2021). Single-strand template repair: Key insights to increase the efficiency of gene editing. Curr. Genet..

[13] Johnston A.D., Simoes-Pires C.A., Suzuki M., Greally J.M. (2019). High-efficiency genomic editing in Epstein-Barr virus-transformed lymphoblastoid B cells using a single-stranded donor oligonucleotide strategy. Commun. Biol..

[14] Guo Q., Mintier G., Ma-Edmonds M., Storton D., Wang X., Xiao X., Kienzle B., Zhao D., Feder J.N. (2018). ‘Cold shock’ increases the frequency of homology directed repair gene editing in induced pluripotent stem cells. Sci. Rep..

[15] Okamoto S., Amaishi Y., Maki I., Enoki T., Mineno J. (2019). Highly efficient genome editing for single-base substitutions using optimized ssODNs with Cas9-RNPs. Sci. Rep..

[16] Schubert M.S., Thommandru B., Woodley J., Turk R., Yan S., Kurgan G., McNeill M.S., Rettig G.R. (2021). Optimized design parameters for CRISPR Cas9 and Cas12a homology-directed repair. Sci. Rep..

[17] Shams F., Bayat H., Mohammadian O., Mahboudi S., Vahidnezhad H., Soosanabadi M., Rahimpour A. (2022). Advance trends in targeting homology-directed repair for accurate gene editing: An inclusive review of small molecules and modified CRISPR-Cas9 systems. Bioimpacts.

[18] Hu J.H., Davis K.M., Liu D.R. (2016). Chemical Biology Approaches to Genome Editing: Understanding, Controlling, and Delivering Programmable Nucleases. Cell Chem. Biol..

[19] Chen S.W., Chen D., Liu B., Haisma H.J. (2022). Modulating CRISPR/Cas9 genome-editing activity by small molecules. Drug Discov. Today.

[20] Ma X.J., Kong L.H., Zhu S.Y. (2017). Reprogramming cell fates by small molecules. Protein Cell.

[21] Baumgard L.H., Hausman G.J., Sanz Fernandez M.V. (2016). Insulin: Pancreatic secretion and adipocyte regulation. Domest. Anim. Endocrinol..

[22] Kleinwort K.J.H., Amann B., Hauck S.M., Hirmer S., Blutke A., Renner S., Uhl P.B., Lutterberg K., Sekundo W., Wolf E. (2017). Retinopathy with central oedema in an INSC94Y transgenic pig model of long-term diabetes. Diabetologia.

[23] Xie B., Wang J., Liu S., Wang J., Xue B., Li J., Wei R., Zhao Y., Liu Z. (2014). Positive correlation between the efficiency of induced pluripotent stem cells and the development rate of nuclear transfer embryos when the same porcine embryonic fibroblast lines are used as donor cells. Cell Reprogram.

[24] Clement K., Rees H., Canver M.C., Gehrke J.M., Farouni R., Hsu J.Y., Cole M.A., Liu D.R., Joung J.K., Bauer D.E. (2019). CRISPResso2 provides accurate and rapid genome editing sequence analysis. Nat. Biotechnol..

[25] Bento A.P., Gaulton A., Hersey A., Bellis L.J., Chambers J., Davies M., Kruger F.A., Light Y., Mak L., McGlinchey S. (2014). The ChEMBL bioactivity database: An update. Nucleic Acids Res..

[26] Milanowska K., Krwawicz J., Papaj G., Kosinski J., Poleszak K., Lesiak J., Osinska E., Rother K., Bujnicki J.M. (2013). REPAIRtoire—A database of DNA repair pathways. FEBS J..

[27] Denes C.E., Cole A.J., Aksoy Y.A., Li G., Neely G.G., Hesselson D. (2021). Approaches to Enhance Precise CRISPR/Cas9-Mediated Genome Editing. Int. J. Mol. Sci..

[28] Yang D., Scavuzzo M.A., Chmielowiec J., Sharp R., Bajic A., Borowiak M. (2016). Enrichment of G2/M cell cycle phase in human pluripotent stem cells enhances HDR-mediated gene repair with customizable endonucleases. Sci. Rep..

[29] Yang H., Ren S., Yu S., Pan H., Li T., Ge S., Zhang J., Xia N. (2020). Methods Favoring Homology-Directed Repair Choice in Response to CRISPR/Cas9 Induced-Double Strand Breaks. Int. J. Mol. Sci..

[30] Maruyama T., Dougan S.K., Truttmann M.C., Bilate A.M., Ingram J.R., Ploegh H.L. (2015). Increasing the efficiency of precise genome editing with CRISPR-Cas9 by inhibition of nonhomologous end joining. Nat. Biotechnol..

[31] Chen X., Zhong S.J., Zhu Y., Dziegielewska B., Ellenberger T., Wilson G.M., MacKerell A.D., Tomkinson A.E. (2008). Rational design of human DNA ligase inhibitors that target cellular DNA replication and repair. Cancer Res..

[32] Song J., Yang D., Xu J., Zhu T., Chen Y.E., Zhang J. (2016). RS-1 enhances CRISPR/Cas9- and TALEN-mediated knock-in efficiency. Nat. Commun..

[33] Li G., Zhang X., Zhong C., Mo J., Quan R., Yang J., Liu D., Li Z., Yang H., Wu Z. (2017). Small molecules enhance CRISPR/Cas9-mediated homology-directed genome editing in primary cells. Sci. Rep..

[34] Paek S.M. (2018). Recent Synthesis and Discovery of Brefeldin A Analogs. Mar Drugs.

[35] Richardson C.D., Kazane K.R., Feng S.J., Zelin E., Bray N.L., Schafer A.J., Floor S.N., Corn J.E. (2018). CRISPR-Cas9 genome editing in human cells occurs via the Fanconi anemia pathway. Nat. Genet..

[36] Moldovan G.L., D’Andrea A.D. (2009). FANCD2 hurdles the DNA interstrand crosslink. Cell.

[37] Killian T., Dickopf S., Haas A.K., Kirstenpfad C., Mayer K., Brinkmann U. (2017). Disruption of diphthamide synthesis genes and resulting toxin resistance as a robust technology for quantifying and optimizing CRISPR/Cas9-mediated gene editing. Sci. Rep..

[38] Aksoy Y.A., Nguyen D.T., Chow S., Chung R.S., Guillemin G.J., Cole N.J., Hesselson D. (2019). Chemical reprogramming enhances homology-directed genome editing in zebrafish embryos. Commun. Biol..

[39] Wienert B., Nguyen D.N., Guenther A., Feng S.J., Locke M.N., Wyman S.K., Shin J., Kazane K.R., Gregory G.L., Carter M.A.M. (2020). Timed inhibition of CDC7 increases CRISPR-Cas9 mediated templated repair. Nat. Commun..

[40] Liu B., Chen S.W., La Rose A., Chen D., Cao F.Y., Zwinderman M., Kiemel D., Aissi M., Dekker F.J., Haisma H.J. (2020). Inhibition of histone deacetylase 1 (HDAC1) and HDAC2 enhances CRISPR/Cas9 genome editing. Nucleic Acids Res..

[41] Kagita A., Lung M.S.Y., Xu H.G., Kita Y., Sasakawa N., Iguchi T., Ono M., Wang X.H., Gee P., Hotta A. (2021). Efficient ssODN-Mediated Targeting by Avoiding Cellular Inhibitory RNAs through Precomplexed CRISPR-Cas9/sgRNA Ribonucleoprotein. Stem Cell Rep..

[42] Hirakawa M.P., Krishnakumar R., Timlin J.A., Carney J.P., Butler K.S. (2020). Gene editing and CRISPR in the clinic: Current and future perspectives. Biosci. Rep..

[43] Hu Q., Lu H., Wang H., Li S., Truong L., Li J., Liu S., Xiang R., Wu X. (2019). Break-induced replication plays a prominent role in long-range repeat-mediated deletion. EMBO J..

[44] Ma X., Chen X., Jin Y., Ge W., Wang W., Kong L., Ji J., Guo X., Huang J., Feng X.H. (2018). Small molecules promote CRISPR-Cpf1-mediated genome editing in human pluripotent stem cells. Nat. Commun..

