# Supplementary material for: Improving the Efficiency of CRISPR Ribonucleoprotein-Mediated Precise Gene Editing by Small Molecules in Porcine Fibroblasts
Source: Animals (Basel). 2024 Feb 25;14(5):719. doi: 10.3390/ani14050719 (PMC10931096; doi:10.3390/ani14050719)
Supplement: Supplementary file 1 [file animals-14-00719-s001.zip › Supplementary Figure S1.pdf]

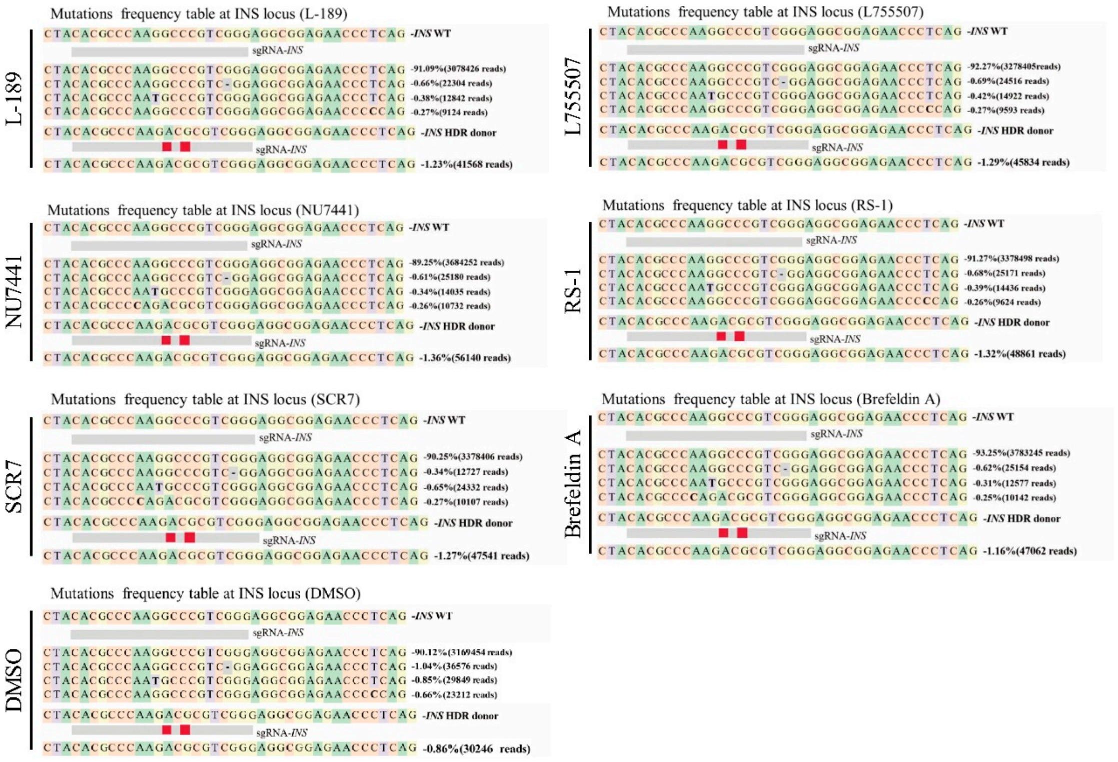

Supplementary Figure S1. The deep sequence results of INS gene precise editing in PFF cells treated with single small molecule L-189, NU7441, SCR7, L755507, RS-1 and Brefeldin A.
