# Supplementary material for: Improving the Efficiency of CRISPR Ribonucleoprotein-Mediated Precise Gene Editing by Small Molecules in Porcine Fibroblasts
Source: Animals (Basel). 2024 Feb 25;14(5):719. doi: 10.3390/ani14050719 (PMC10931096; doi:10.3390/ani14050719)
Supplement: Supplementary file 1 [file animals-14-00719-s001.zip › Supplementary Figure S2.pdf]

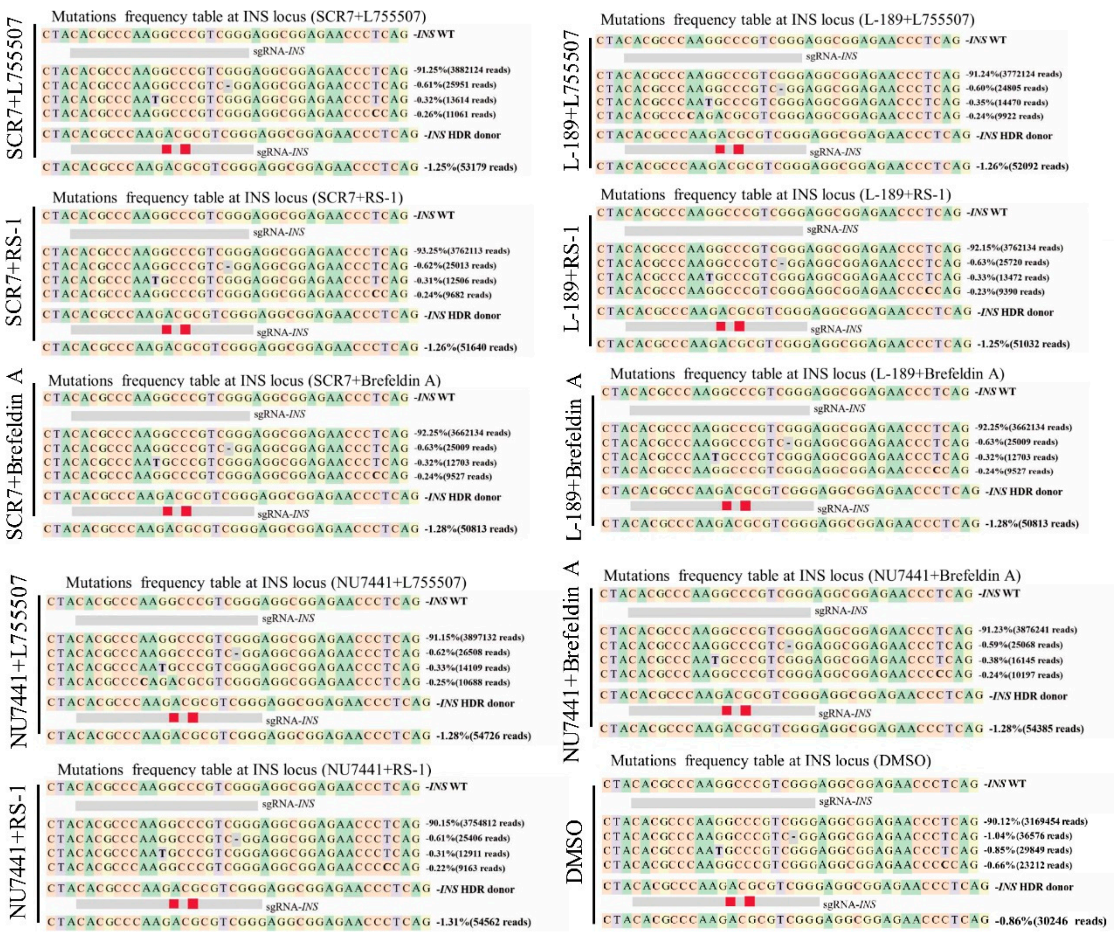

Supplementary Figure S2. The deep sequence results of INS gene precise editing in PFF cells treated with the combination of two small molecules, L-189+L755507, L-189+RS-1, L-189+BrefeldinA, SCR7+L755507, SCR7+RS-1, SCR7+BrefeldinA, NU7441+L755507, NU7441+RS-1, and NU7441+Brefeldin A.
