# Supplementary material for: Improving the Efficiency of CRISPR Ribonucleoprotein-Mediated Precise Gene Editing by Small Molecules in Porcine Fibroblasts
Source: Animals (Basel). 2024 Feb 25;14(5):719. doi: 10.3390/ani14050719 (PMC10931096; doi:10.3390/ani14050719)
Supplement: Supplementary file 1 [file animals-14-00719-s001.zip › Supplementary Table S1. ssODN synthesized in this paper.pdf]

Supplementary Table S1. ssODN synthesized in this paper

Gene (Architecture)	Sequence(5’->3’)
INS（ssODN-INSA54T-M）	ACGCCCCCTCGGCTCACCCTGAGGGTTCTCCGCCTCCCGACGCGTCTTGGGCGTGTAGAAGAAGCCGCGCTCCCCGCACACCAGGTACAGCGCCTCCACCAGGTGGGAGCCGCACA
